# In‐utero exposure to tenofovir‐containing pre‐exposure prophylaxis and bone mineral content in HIV‐unexposed infants in South Africa

**DOI:** 10.1002/jia2.26379

**Published:** 2024-11-11

**Authors:** Kerusha Reddy, Kimesh L. Naidoo, Carl Lombard, Zukiswa Godlwana, Alicia C. Desmond, Richard Clark, James F. Rooney, Glenda Gray, Dhayendre Moodley

**Affiliations:** ^1^ Department of Paediatrics and Child Health School of Clinical Medicine University of KwaZulu Natal Durban South Africa; ^2^ King Edward VIII Hospital Durban South Africa Durban South Africa; ^3^ Biostatistics Unit South African Medical Research Council Tygerberg South Africa; ^4^ Division of Epidemiology and Biostatistics Department of Global Health University of Stellenbosch Tygerberg South Africa; ^5^ Centre for the Program of AIDS Research in South Africa (CAPRISA) Durban South Africa; ^6^ Gilead Sciences Inc Foster City California USA; ^7^ South African Medical Research Council Cape Town South Africa; ^8^ Department of Obstetrics and Gynaecology School of Clinical Medicine University of KwaZulu Natal Durban South Africa

**Keywords:** tenofovir disoproxil fumarate, pre‐exposure prophylaxis, in‐utero exposure, bone mineral content, breastfeeding, infants

## Abstract

**Introduction:**

Tenofovir disoproxil fumarate (TDF) is a common drug of choice for pre‐exposure prophylaxis (PrEP) or as a combination HIV treatment for pregnant women. In‐utero exposure to TDF was found to be associated with lower bone mineral content (BMC) in HIV‐exposed uninfected neonates. Data for infants born to women taking TDF‐PrEP are lacking. The CAP016 randomized control trial was conducted in South Africa between September 2017 and August 2021 and pregnant women either initiated TDF/FTC PrEP in pregnancy (Immediate PrEP arm‐IP) or at cessation of breastfeeding (Deferred PrEP arm‐DP). In a secondary data analysis, we evaluated BMC in HIV‐unexposed infants in the CAP016 trial in the first 18 months of life in association with maternal TDF‐PrEP use during pregnancy.

**Methods:**

Infants born to women randomized to the IP arm or DP arm in the CAP016 clinical trial had BMC measurements of the whole body with head (WBH) and lumbar spine (LS) by dual energy X‐ray absorptiometry (DXA) at 6, 26, 50 and 74 weeks.

**Results:**

Of 481 infants born to women enrolled in the CAP016 clinical trial, 335 (69.6%) infants had a minimum of one DXA scan of the WBH and LS between 6 and 74 weeks of age (168 IP and 167 DP). Women in the IP arm received TDF‐FTC PreP for a median of 19 weeks between initiation in pregnancy and delivery. Using a mixed linear regression model and adjusted for gestational age, sex and ever‐breastfed, the mean difference (95% CI) for BMC of the WBH between IP and DP arms were −0.74 (−8.69 to 7.20), −1.26 (−10.75 to 8.23), −9.17 (−20.02 to 1.69) and 5.02 (−6.74 to 16.78) g at 6, 26, 50 and 74 weeks (*p* = 0.283). Mean differences in BMC of the LS were 0.07 (−0.10 to 0.23), 0.02 (−0.18 to 0.22), −0.14 (−0.36 to 0.09) and 0.14 (−0.11 to 0.38) g at 6, 26, 50 and 74 weeks, respectively (*p* = 0.329).

**Conclusions:**

In a randomized controlled trial, there were no differences in BMC of the WBH and LS between infants exposed to in‐utero TDF‐FTC PrEP and unexposed infants in the first 18 months of life.

## 1 INTRODUCTION

Oral pre‐exposure prophylaxis (PrEP) consisting of tenofovir disoproxil fumarate (TDF) and emtricitabine (FTC) is now widely accessible as an effective biomedical intervention for populations at high risk of acquiring HIV, including pregnant women [1, 2]. Oral TDF/FTC PrEP remains the only available biomedical prevention method for pregnant and lactating women until studies of other biomedical interventions generate reassuring pregnancy safety data similar to that of TDF/FTC.

TDF, a prodrug of tenofovir (TFV), is generally safe, is an effective antiretroviral and is widely used in combination with other antiretrovirals in the treatment of people living with HIV. However, through several clinical studies, TDF was associated with reduced renal function through its direct effect on proximal tubular excretion [3, 4]. In addition, HIV treatment studies have also demonstrated an association between TDF and decreased bone mineral density in adults [5]. The biological mechanisms for how TDF affects bone integrity are unclear. Early animal studies suggest that TDF decreases extracellular adenosine concentration which in turn is associated with an increase in osteoclast differentiation and subsequent bone loss [6]. Other postulated mechanisms include TDF's effect on the parathyroid hormone, a regulator of vitamin D metabolism, and TDF may also have an indirect effect on bone through its proximal renal tubule toxicity and accompanying phosphate loss [7, 8].

While TDF has been independently associated with bone loss in adults [5], lower bone mineral density and associated fractures are reportedly more common in people living with HIV than people without HIV, suggesting that HIV proteins may also have suppressive effects on osteoblastic activity [9]. However, TDF used in combination with FTC as PrEP in young healthy African women was also found to be associated with a decline in bone mineral density (BMD) by 48 weeks of use [10]. While the BMD decline in adults living with HIV was associated with more bone fractures, the clinical implications of bone decay in PrEP users appear non‐significant [11].

TDF, when ingested, is first converted to TFV in the plasma and gut, and then intracellularly phosphorylated to tenofovir‐diphosphate (TFV‐DP), an active form of TFV [12, 13]. TDF is known for its high placental transfer rate with infant‐maternal TFV‐DP ratios ranging between 0.60 and 0.88 or even higher when hair samples were tested [14, 15]. Given the direct effect of TDF on osteoclast and osteoblast activity and since much of foetal bone development occurs in the second half of pregnancy, several studies have explored the effect of in‐utero TDF exposure on neonatal and infant bone mineral content (BMC) or bone mineral density. These studies have mostly included infants born to women living with HIV and receiving combination antiretroviral treatment during pregnancy, and not all studies concurred in finding an association between TDF and infant bone loss [16−18]. Several factors could have contributed to these inconsistent interpretations. Firstly, although infants were HIV uninfected, they were HIV‐exposed and infants were also exposed to other antiretrovirals also known to be associated with bone mineralization [19, 20].

Studies determining the independent effect of in‐utero TDF exposure on BMD and BMC of HIV‐unexposed infants born to women taking TDF‐containing PrEP are sparse. In a secondary endpoint analysis of an open‐label randomized controlled trial assessing the safety of TDF/FTC PrEP initiated in the second trimester of pregnancy [21], we evaluated changes in BMC in the infants using dual energy X‐ray absorptiometry (DXA) scans of the whole body (with head) and lumbar spine (LS) at multiple points in the first year of life in association with in‐utero exposure to TDF.

## 2 METHODS

### 2.1 Population and study design

The CAP016 trial was an open‐label randomized control trial conducted in a peri‐urban setting in South Africa where pregnant women either initiated TDF/FTC PrEP in the second trimester of pregnancy (IP arm) or at cessation of breastfeeding (DP arm). The results of the primary safety endpoint analysis of pregnancy and neonatal outcomes have been published [21]. In the CAP016 study, mother‐baby pairs were followed up for up to 18 months post‐delivery. The purpose of this secondary analysis is to describe the effect of maternal TDF/FTC PrEP use during pregnancy on the infant's bone health by comparing BMC of the whole body (with head) and LS between infants in the IP and DP arms. During pregnancy, we collected clinical data, adherence data and blood samples from all women at their scheduled 4‐weekly antenatal visits. We repeated the data collection that included breastfeeding practice for mother‐baby pairs at birth, 6 weeks, 6 months, 12 months and 18 months. Data for this secondary analysis were collected between September 2017 and August 2021.

### 2.2 Maternal and clinical data

Following delivery, the clinical characteristics of infants were extracted from the neonatal record viz. birth weight and length, head circumference, gestational age by modified Ballard score (done by the research staff and attending clinician) and gestational age (calculated by ultrasound and by dates as determined by last normal menstrual period). Infants had scheduled follow‐up visits at 6, 26, 50 and 74 weeks of age. At these visits, the growth parameters (weight, length and head circumference) of these infants were recorded and plotted on standard infant growth charts. Infant feeding modality was recorded at each visit (cessation of breastfeeding is defined as 28 days after last exposure to breast milk).

### 2.3 Dual‐energy X‐ray absorptiometry

Infants in IP and DP arms had DXA scans of the whole body including the head (WBH) and LS on a HOLOGIC Discovery Wi model (S/N 84656) with Hologic APEX Software Version 3.1.2 at various time points in the 18‐month follow‐up. All scans were performed by an experienced well‐trained radiographer on site. Scans were printed and BMC and BMD were subsequently captured in the CAP016 parent database. Based on the timing of DXA scans, all scans were also classified as early (6 or 26 weeks) or late scans (50 or 74 weeks).

### 2.4 Measure of in‐utero TDF exposure

Studies comparing self‐reports and pill‐counts to TFV‐DP drug levels as measures of adherence in early PrEP clinical trials revealed over‐reporting of adherence for several patient‐centred reasons [22, 23]. For our post‐hoc secondary per‐protocol analysis, dried blood spot (DBS) samples that were collected from mothers in the IP arm at 4 and 12 weeks post‐randomization and stored for later evaluation were tested for TFV‐DP level. DBS assays report drug concentrations in femtomoles (fmol) measured in a 3 mm disc punched from the dried blood on the card. Extractions from a 50‐µl DBS were tested for TFV‐DP using a validated high‐performance liquid chromatography–tandem mass spectrometry (LC‐MS/MS) method at the University of Cape Town, South Africa [24]. The lower limit of quantification for TFV‐DP is 16.6 fmol/3‐mm punch. A correction factor was applied for the TFV‐DP concentration in ng/ml to be converted to fmol/3‐mm punch, for comparison across studies using this standard unit of measure of TFV‐DP [24]. TFV‐DP levels were measured at two time points during pregnancy, 4 and 12 weeks post randomization, taking an average between these two visits for our exploratory analysis

### 2.5 Data analysis

Descriptive statistics such as means, standard deviations, frequencies and percentages were calculated for mothers and infants in the IP and DP arms. Birth characteristics were compared between randomization arms using *t*‐tests for numeric data and Chi‐square tests or Fisher's exact test (small frequencies) for categorical data. Student's *t*‐tests assuming unequal variances were used to compare mean BMC at weeks 6, 24, 50 and 74 weeks between both groups as an unadjusted analysis. In the primary analysis of the CAP016 data, in‐utero exposure to TDF‐containing PrEP was not associated with gestational age at birth and preterm births [21]. Gestational age at birth, however, has been independently associated with bone mineralization in newborns, thus preterm infants have a lower BMC than term infants [25]. For the adjusted analysis, a mixed linear regression model of whole‐body with head and LS BMC on week, gestational age, sex, ever breastfed and maternal TDF exposure status (IP and DP arms) were used. The interaction between week and randomization arm was used as the overall test for the difference in the mean BMC profiles of the infants. To take account of the repeated BMC measurements over time, infants were specified as the random effect and consisted of intercepts only. Time‐specific mean differences between the IP and DP arms were estimated and reported with 95% CI.

In an ad‐hoc analysis, and applying the adherence benchmarks from a directly observed pharmacokinetic modelling study of TDF/FTC PrEP in pregnant and postpartum women, the maternal TFV‐DP levels in our study were categorized as undetectable, <200, 200−599 and >600 fmol/punch [26]. These categories predict the number of doses taken in a week that is <200 fmol/punch equivalent to less than 2 doses/week, 200−599 fmol/punch equivalent to 2−6 doses/week and >600 fmol/punch equivalent to an estimated 7 doses/week. We compared BMC of the WBH and LS in infants in the IP arm whose mothers demonstrated moderate to high (>200 fmol/punch) adherence to oral PrEP during pregnancy with infants in the DP arm.

### 2.6 Ethical consideration

The parent study (CAP 016) was conducted in compliance with approval from the South African Health Products Regulatory Authorities (SAHPRA) and the University of KwaZulu‐Natal Biomedical Research Ethics Committee (BFC243/16). All maternal participants signed an informed consent that included infant follow‐up procedures and serial DXA scans.

### 2.7 Participant confidentiality

All laboratory specimens, evaluation forms, reports and other records that are transferred or transmitted off‐site for processing were identified only by a coded number to maintain subject confidentiality. All records were kept in a secured area with access limited to authorized personnel only.

## 3 RESULTS

**Figure 1:**
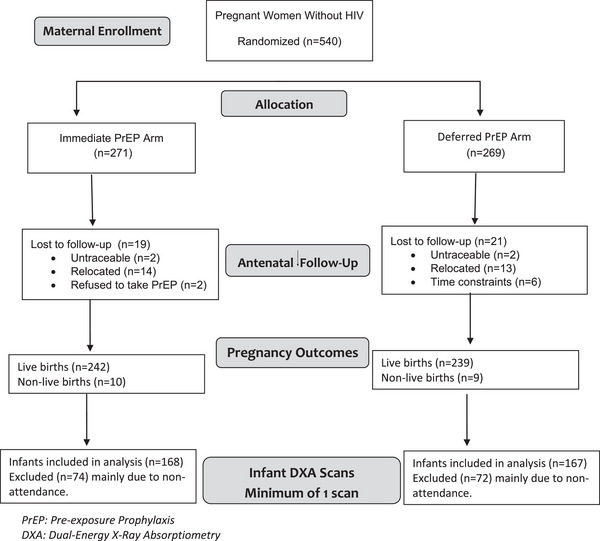
Longitudinal evaluation of infants born to women randomized to the immediate pre‐exposure prophylaxis arm or deferred pre‐exposure prophylaxis arm.

Of the 481 infants born to women enrolled in the CAP016 clinical trial, 168 infants in the IP arm and 167 in the DP arm returned for one or more scheduled visits between 6 and 74 weeks and 107 (64.0%) and 105 (62.9%) infants had two or more DXA scans in the IP and DP arms, respectively (Figure 1).

### 3.1 Birth characteristics of infants: comparison between immediate PrEP and deferred PrEP arms

There were 19 infants born <37 weeks of gestation; the proportion of preterm births across both groups was not significantly different (Table 1). Infants in the IP and DP arms did not differ in birth weight, sex distribution, birth length and breastfeeding exposure (Table 1).

The proportion of infants breastfed beyond 7 days of age was similar across both groups (84.5% vs. 85.6%) and the median duration of breastfeeding was 10 (IQR 4; 22) and 14 (IQR 4; 32) weeks in the IP and DP arms, respectively (*p* = 0.178). Timing and frequency of DXA scans during the 74‐week follow‐up period did not differ between IP and DP arms (Table 1). In addition to the 146 infants who did not return for follow‐up, an additional 22 infants who did return for follow‐up but did not have evaluable DXA results (14 in the IP arm and 8 in the DP arm) were excluded from this secondary analysis. There were no significant differences in birth weight (*p* = 0.766), length (*p* = 0.457), head circumference (*p* = 0.480) and gestational age at birth (*p* = 0.134) between infants who had a DXA scan done, infants who did not return for follow‐up and infants with no evaluable DXA results.

**Table 1:** Infant characteristics by maternal randomization arm

Characteristics	Immediate PrEP (*N* = 168)	Deferred PrEP (*N* = 167)	*p* value
**Sex *n* (%)**			
*Missing data*	1	2	0.827
Male	86 (51.2)	87 (52.1)	
Female	81 (48.2)	78 (46.7)	
**Birth weight, g** (*n*)			
*Missing data*	2	0	0.399
Mean (SD)	3144 (536)	3102 (465)	
**Low birth weight (<2500 g) (*n* [%])**			
Yes	16 (9.5)	12 (7.2)	0.437
No	150 (89.3)	155 (92.8)	
**Birth length, cm (*n*)**	139	132	
*Missing data*	29	35	0.351
Mean (SD)	49.4 (3.3)	49.2 (3.6)	
**Gestational age at birth, weeks** (*n*)	168	167	
Mean (SD)	38.9 (1.7)	39.1 (1.8)	0.462
**Preterm birth (<37 weeks)** *n* (%)			
Yes	11 (6.5)	8 (4.8)	0.638
No	157 (93.5)	159 (95.2)	
**Breastfeeding** (*n* [%])			
More than 7 days	142 (84.5)	143 (85.6)	0.878
0 to < 7 days	26 (15.5)	24 (14.4)	
**Total number of DXA scans**	363	332	–
**Frequency of DXA scans *n* (%)**			0.458
Early only (6 or 26 weeks)	77 (45.8)	75 (44.9)	
Late only (50 or 74 weeks)	11 (6.6)	17 (10.2)	
Early and late	80 (47.6)	75 (44.9)	

### 3.2 BMC of the whole body (with head) and lumbar spine by randomization arm

**Figure 2:**
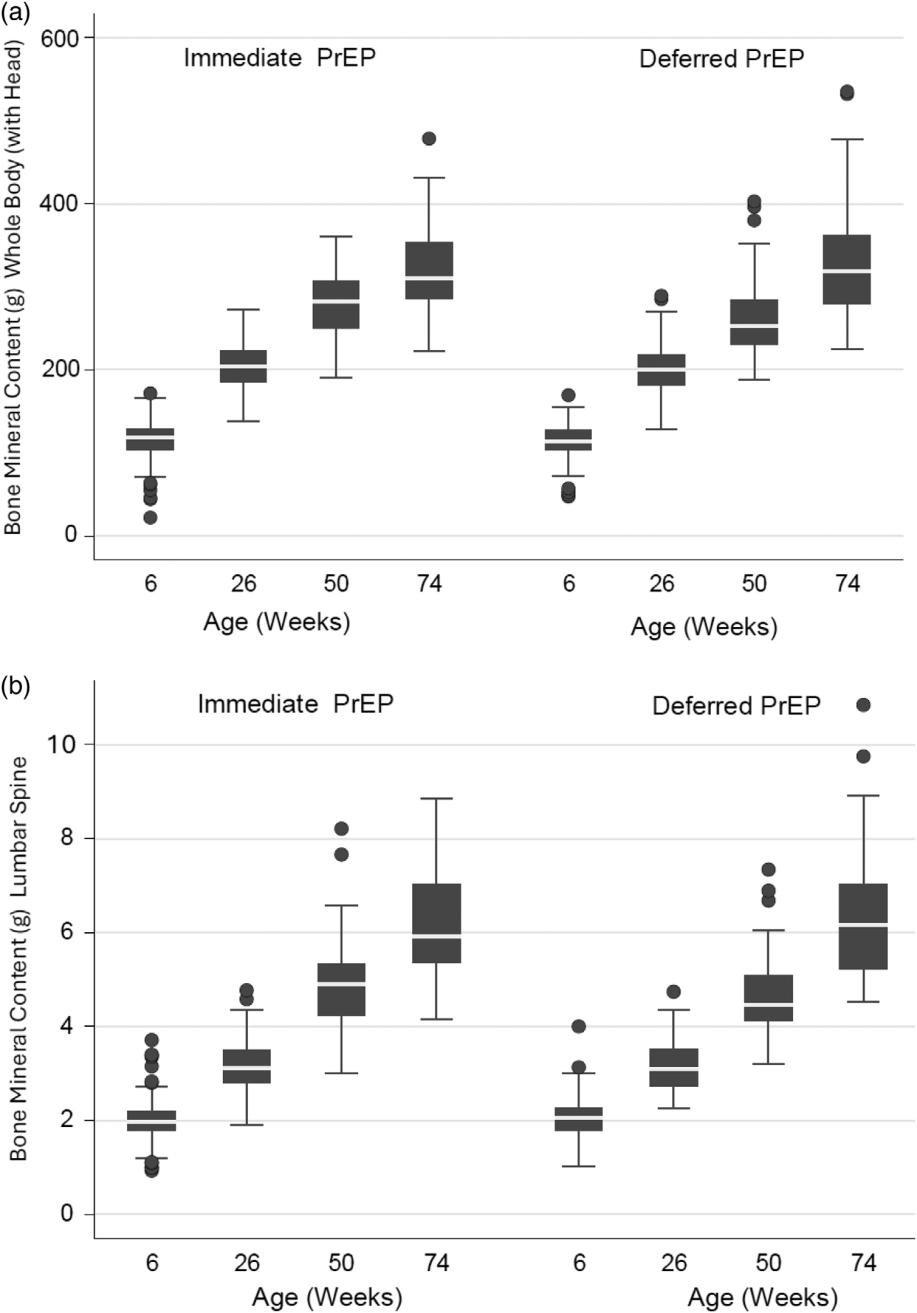
Box plot of unadjusted mean (SD) of whole‐body with head (A) and lumbar spine (B) bone mineral content (BMC) (g) from dual energy X‐ray absorptiometry by randomization arm.

Absolute values for BMC (g) of the whole body (with head) for each visit (age in weeks) for infants in the IP and DP arms are presented in a Box plot (Figure 2A). At 6, 26, 50 and 74 weeks, the median (IQR) of BMC (g) of the WBH were 119.06 (103.0; 128.9), 204.46 (185.6; 223.8), 282.76 (249.6; 307.7) and 310.26 (285.7; 354.4), respectively. The median (IQR) for BMC in infants in the DP arm were 114.10 (103.6; 127.9), 200.23 (181.3; 217.9), 253.33 (230.4; 284.1) and 319.03 (279.8; 361.8) at 6, 26, 50 and 74 weeks, respectively.

Absolute values for BMC (g) of the LS for each visit (age in weeks) for infants in the IP and DP arms are presented in Figure 2B. At 6, 26, 50 and 74 weeks, the median (IQR) of BMC (g) of the LS were 1.97 (1.8; 2.2), 3.12 (2.8; 3.5), 4.91 (4.2; 5.3) and 5.92 (5.4; 7.0), respectively. The median (IQR) for LS BMC in infants in the DP arm were 2.05 (1.8; 2.3), 3.08 (2.7; 3.5), 4.46 (4.1; 5.1) and 6.16 (5.2; 7.0) at 6, 26, 50 and 74 weeks, respectively.

### 3.3 Adjusted difference in BMC of the whole body (with head) and lumbar spine by randomization arm

From the linear regression model, the 6‐week estimated adjusted mean whole‐body BMC (with head) was 0.74 g lower (95% CI −8.69 to 7.20; *p* = 0.854) in the IP arm compared to the DP arm. The mean difference increased at 26 and 50 weeks when the mean WBH BMC was 1.26 and 9.17 g lower in the IP arm compared to the DP arm, respectively. These differences were not significant (Table 3). Overall the mean (SE) BMC (g) of the WBH after adjusting for gestational age, sex and breastfed for more than 7 days was not significantly different between treatment arms over the 18 months (*p* = 0.283).

The adjusted mean LS BMC was 0.07 and 0.02 g higher (95% CI −0.10 to 0.23; −0.18 to 0.22) in the IP arm compared to the DP arm at 6 and 26 weeks, respectively. Overall the mean (SE) BMC (g) of the LS after adjusting for gestational age, sex and ever breastfed was not significantly different between treatment arms over the 18‐month period (*p* = 0.329) (Table 2).

**Table 2:** Mean (SE) of bone mineral content (g) of the whole body (with head) and lumbar spine in infants in the immediate PrEP arm in comparison to infants in the deferred PrEP arm (adjusted for gestational age, sex and ever breastfed)

	Number (*n*)	Immediate PrEP mean (SE)	Number (*n*)	Deferred PrEP mean (SE)	Mean difference (95% CI)	Adjusted *p* value
	Whole body (with head) bone mineral content (g)					
Age (weeks)						0.283
6	137	115.11 (2.90)	128	115.86 (2.82)	−0.74 (−8.69 to 7.20)	0.854
26	100	200.92 (3.60)	80	202.18 (3.23)	−1.26 (−10.75 to 8.23)	0.795
50	66	266.24 (3.94)	65	275.41 (3.89)	−9.17 (−20.02 to 1.69)	0.098
74	56	327.74 (4.30)	55	322.72 (4.20)	5.02 (−6.74 to 16.78)	0.403
	Lumbar spine bone mineral content (g)					
Age (weeks)						0.329
6	131	2.06 (0.06)	126	1.99 (0.06)	0.07 (−0.10 to 0.23)	0.442
26	98	3.13 (0.08)	78	3.11 (0.07)	0.02 (−0.18 to 0.22)	0.838
50	66	4.70 (0.08)	66	4.83 (0.08)	−0.14 (−0.36 to 0.09)	0.241
74	58	6.29 (0.09)	55	6.15 (0.09)	0.14 (−0.11 to 0.38)	0.274

### 3.4 BMC (g) of the whole body (with head) and lumbar spine by maternal adherence to PrEP in the IP arm versus DP arm

The median (IQR) period of PrEP use among women in the IP arm was 19 (15; 23) weeks during pregnancy. The median (IQR) TFV‐DP level in pregnancy was 281 (51; 535) fmol/punch. Of the 167 women in the IP arm, 22 (13.2%) women had undetectable TVF‐DP, while 48 (28.7%), 67 (40.1%) and 30 (18.0%) women had TFV‐DP levels <200, 200−599 and ≥ 600 fmol/punch, respectively. Overall 97 (58.1%) women in the IP arm demonstrated moderate to high adherence to their daily oral PrEP regimen during pregnancy.

The mean difference (95% CI) in BMC of the LS and WBH between infants born to women with moderate‐to‐high adherence to oral PrEP in the IP arm and infants in the DP arm were not significant at all visits between 6 and 74 weeks (Table 3). BMC of the WBH and LS were much lower in infants in the Moderate‐to‐High Adherence group at 74 weeks when compared to the infants in the DP arm. However, this remained non‐significant probably due to the small number of infants seen at 74 weeks.

**Table 3:** Whole body (with head) and lumbar spine bone mineral content in infants by randomization arm and maternal PrEP adherence

	Immediate PrEP arm		Deferred PrEP arm	Mean difference (95% CI): moderate‐to‐high adherence versus deferred PrEP	*p* value
Age (weeks)	Low adherence (<200 fmol/punch)	Moderate‐to‐high adherence (≥200 fmol/punch)	PrEP unexposed		
**Bone mineral content at lumbar spine (g)**					
6	*n* = 72 1.66 (0.86)	*n* = 92 1.52 (0.95)	*n* = 155 1.68 (0.89)	−0.16 (−0.39 to 0.08)	0.187
26	*n* = 41 3.21 (0.53)	*n* = 56 3.12 (0.52)	*n* = 79 3.13 (0.51)	−0.01 (−0.19 to 0.17)	0.910
50	*n* = 27 5.07 (1.05)	*n* = 39 4.75 (0.79)	*n* = 66 4.65 (0.83)	0.09 (−0.23 to 0.43)	0.551
74	*n* = 26 6.24 (1.08)	*n* = 32 5.99 (1.13)	*n* = 55 6.33 (1.36)	−0.34 (−0.90 to 0.23)	0.242
**Bone mineral content of whole body (with head) (g)**					
6	*n* = 72 97.20 (45.58)	*n* = 93 92.46 (50.48)	*n* = 155 95.12 (47.06)	−2.67 (−15.16 to 9.83)	0.675
26	*n* = 42 202.76 (32.87)	*n* = 57 205.14 (26.06)	*n* = 81 201.37 (29.37	3.77 (−5.82 to 13.36)	0.439
50	*n* = 27 291.85 (38.71)	*n* = 39 269.31 (38.54)	*n* = 65 264.0 (44.93)	5.31 (−11.83 to 22.45)	0.540
74	*n* = 26 328.54 (56.78)	*n* = 30 314.05 (49.90)	*n* = 55 331.05 (67.54)	−17.01 (−44.97 to 10.96)	0.230

## 4 DISCUSSION

In an intent‐to‐treat analysis, and after adjusting for gestational age, infant sex and breastfeeding for more than 7 days, infants born to women who initiated TDF‐FTC PrEP in the second trimester of pregnancy did not have significant changes in BMC of the LS and whole body (with head) between birth and 74 weeks of age when compared to infants born to women who did not initiate oral PrEP during pregnancy (DP arm). In addition, in an exploratory post‐hoc analysis and using maternal TFV‐DP levels as a benchmark for adherence to the daily oral PrEP regimen, mean differences in BMC of the LS and WBH between infants born to women who demonstrated a moderate to high adherence (TFV‐DP > 200 fmol/punch) to oral PrEP in pregnancy and infants in the DP arm remained non‐significant.

The effect of in‐utero exposure to maternal TFV‐based antiretroviral treatment on bone health of HIV‐exposed infants has previously been studied. Still, it remains controversial due to variations in combination antiretroviral treatment investigated in these few studies, exposure to HIV and duration of infant monitoring. In the PHACS‐SMARTT study, Siberry et al. assessed whole‐body BMC in HIV‐exposed infants at birth only and reported significantly lower BMC in infants exposed to in‐utero TDF versus those with no TDF exposure [17]. In a recently published sub‐study of the PROMISE trial, whole‐body BMC in the neonatal period was significantly lower in infants whose mothers received either a TDF‐based antiretroviral treatment (ART) or a non‐TDF‐based ART when compared to no maternal ART suggestive of other factors affecting infant BMC including the use of a protease inhibitor (PI)‐containing regimen [19]. Furthermore, BMC of the LS was not significantly different between any of the study arms. In a pilot study of HIV/hepatitis B‐coinfected pregnant women, BMD and BMC were assessed at age 6 months of life in 14 TFV‐exposed and 13 unexposed infants [18]. The authors observed a trend towards lower BMC and BMD in infants exposed to maternal TDF but noted these were not statistically significant. Excluding HIV exposure, children of hepatitis B virus (HBV)‐infected mothers who did or did not receive TDF treatment during late pregnancy had comparable BMD of the LS and left hip at a median age of 4 years, suggesting no effect of in‐utero TDF exposure on long‐term bone health [27].

Our study is the first to report LS and whole‐body BMC in infants born to women not living with HIV and randomized to receive oral TDF/FTC PrEP during pregnancy versus delayed TDF/FTC PrEP use until breastfeeding cessation. In addition, we compared BMC between in‐utero PrEP exposed (IP arm) and unexposed (DP arm) infants at more than one time point between 6 and 74 weeks of age. The lack of significant differences in BMC of LS and WBH between infants exposed to in‐utero TDF‐containing PrEP versus no exposure to in‐utero TDF‐PrEP at all time points from 6 to 74 weeks is consistent with findings from the only other study of HIV‐unexposed infants and indeed very reassuring [28]. Wu et al. reported no difference between mean whole‐body BMD at 36 months of age among 40 children with and 71 without in‐utero PrEP exposure. They further concluded that TDF‐PrEP exposure was not associated with BMD or height at 36 months [28].

In adult PrEP studies, the proportion of random samples with undetectable plasma TFV was estimated to be around 50% [29]. In a recent modelling study, the authors found that the variability in efficacy outcomes across studies can be explained by the proportion of trial participants not taking the prescribed drugs [29]. Likewise, inconsistent safety outcomes in PrEP studies can also be explained by variations in adherence to the daily oral regimen. Our post‐hoc exploratory per‐protocol analysis revealed a slightly higher proportion (87%) of pregnant women with detectable TFV‐DP levels, although more than 50% demonstrated a moderate to high adherence (>200 fmol/punch). Our infant bone integrity data in the latter group of women was reassuringly similar to infants never exposed to in‐utero TDF.

Clinical implications of compromised bone integrity as a result of TDF exposure are of public health importance. Fragility bone fractures associated with compromised bone integrity are more commonly seen in older adults living with HIV and on long‐term combination antiretroviral treatment [30, 31]. In children exposed to TDF, no such reports of fragility fractures are yet available. However, the rate of bone mineralization in children could manifest itself in abnormal growth development. In a subsequent analysis of the PHACS‐SMARTT study, the authors reported that while growth parameters were similar at birth, at 1 year of age, those with in‐utero TDF exposure had a slightly lower mean length and head circumference, but their weights were similar, suggestive of clinical implications of lower BMC at birth [32]. Our evaluation of infant growth metrics for our study population has been submitted as a separate manuscript and is under review. Briefly, mean length‐for‐age z‐score (LAZ) scores were consistently low across both groups of infants with in‐utero exposure to TDF or Deferred PrEP arm from 6 weeks through to 74 weeks. Despite in‐utero exposure to high levels of TFV‐DP, our findings are reassuringly suggestive that in‐utero exposure to TDF‐PrEP may not be associated with poor bone integrity in infancy as supported by other studies including HIV treatment studies [14, 28].

Our study has several strengths. This was a secondary data analysis from a randomized controlled study with the Deferred PrEP arm serving as the control. This is also the first longitudinal evaluation with multiple points of assessment from birth to 18 months. We also used an objective measure of assessment of adherence by measuring TFV‐DP levels at two points in pregnancy. Unfortunately, our study is not without its limitations. Follow‐up of infants in the CAP016 clinical trial occurred during the COVID pandemic resulting in a very high attrition rate. As a result, we were unable to determine a percentage change in BMC in infants from 6 weeks to 18 months due to the small sample who completed the study.

## 5 CONCLUSIONS

Our findings are reassuringly suggestive that in‐utero TDF exposure does not alter bone integrity among breastfed infants in the first 18 months of infancy even among women who demonstrate moderate to high adherence to the oral PrEP regimen in pregnancy.

## COMPETING INTERESTS

JFR and RC declare that they are employees of Gilead Sciences Inc and received stock options as part of their compensation packages. All other authors declare no competing interests.

## AUTHORS’ CONTRIBUTIONS

KR, KLN and DM conceptualized the study, provided oversight of the formal analysis of study data and wrote the first draft of the manuscript.

CL conducted the formal analysis of the study data.

JFR, RC and GG provided financial support and resources for the project and assisted with the design of the research.

ZG and ACD played a key role in the conduct of the research and data collection.

All authors reviewed, commented on and approved this manuscript before submission for publication.

## FUNDING

The study was supported by the South African Medical Research Council and Gilead Sciences, Inc. Investigational product was provided by Gilead Sciences, Inc.

## CLINICAL TRIAL NUMBER

CAP016 is on ClinicalTrials.gov, with tracking number NCT3227731 (https://clinicaltrials.gov/ct2/show/NCT03227731)

## Data Availability

Anonymized participant data will be made available upon request directed to the corresponding author.
